# Supplementary material for: End Sequence Analysis Toolkit (ESAT) expands the extractable information from single-cell RNA-seq data
Source: Genome Res. 2016 Oct;26(10):1397–410. doi: 10.1101/gr.207902.116 (PMC5052061; doi:10.1101/gr.207902.116)
Supplement: Supplemental Material [file supp_gr.207902.116_Supp_Materials.tar.gz › ESAT-esat_v0.1/doc/html/classjaligner_1_1_directions.html]

ESAT: jaligner.Directions Class Reference


ESAT


- Main Page
- Related Pages
- Packages
- Classes
- Files

- Class List
- Class Index
- Class Hierarchy
- Class Members

All Classes Namespaces Files Functions Variables Enumerator Pages

- jaligner
- Directions

Static Public Attributes |
List of all members

jaligner.Directions Class Referenceabstract

|  |  |
| --- | --- |
| Static Public Attributes | |
| static final byte | STOP = 0 |
|  | |
| static final byte | LEFT = 1 |
|  | |
| static final byte | DIAGONAL = 2 |
|  | |
| static final byte | UP = 3 |
|  | |

## Detailed Description

Traceback directions.

Author
:   Ahmed Moustafa (ahmed.nosp@m.@use.nosp@m.rs.sf.nosp@m..net)

## Member Data Documentation

final byte jaligner.Directions.DIAGONAL = 2

static

Traceback direction diagonal

final byte jaligner.Directions.LEFT = 1

static

Traceback direction left

final byte jaligner.Directions.STOP = 0

static

Traceback direction stop

final byte jaligner.Directions.UP = 3

static

Traceback direction up

---

The documentation for this class was generated from the following file:

- java/jaligner/Directions.java


---

Generated on Fri Aug 15 2014 15:11:05 for ESAT by  

 1.8.7
